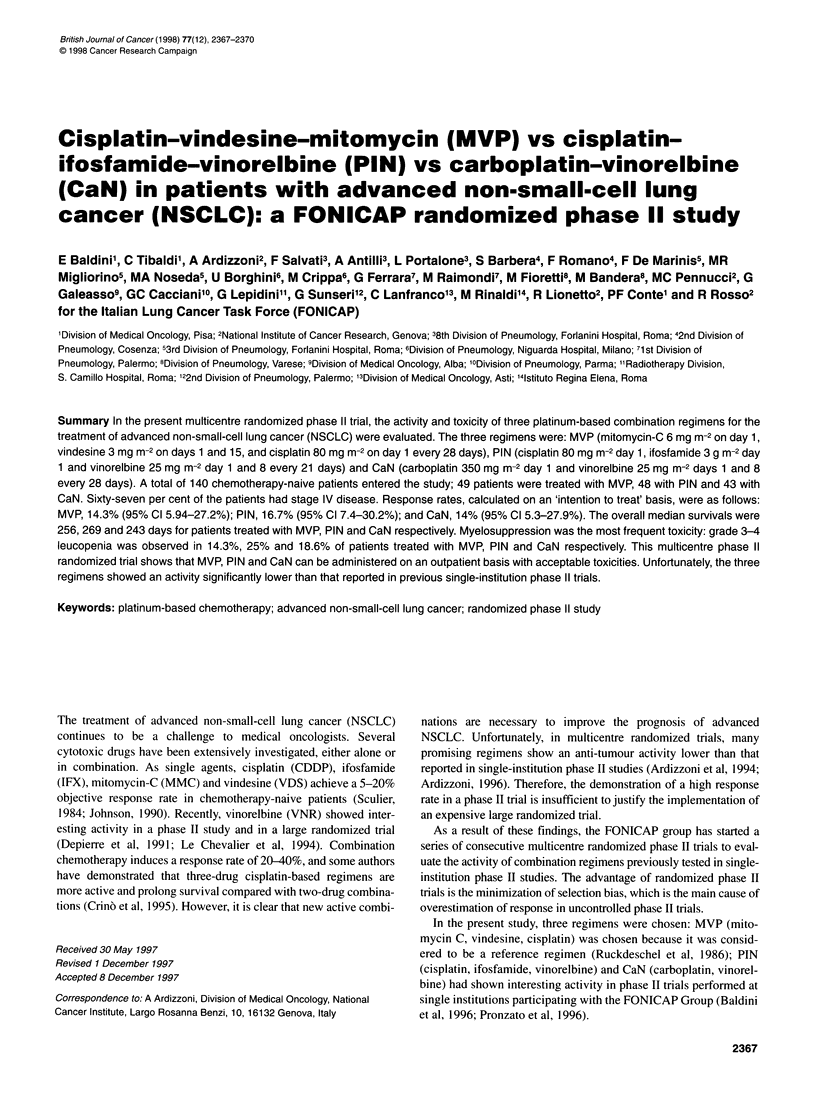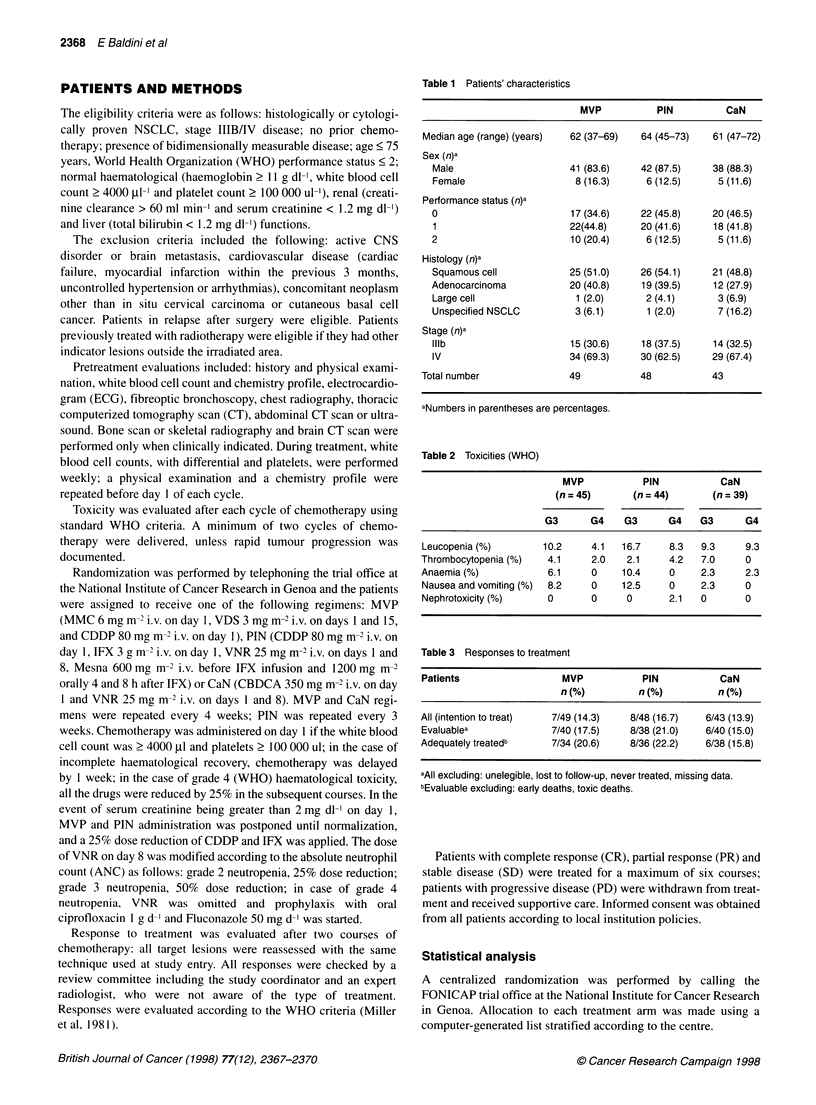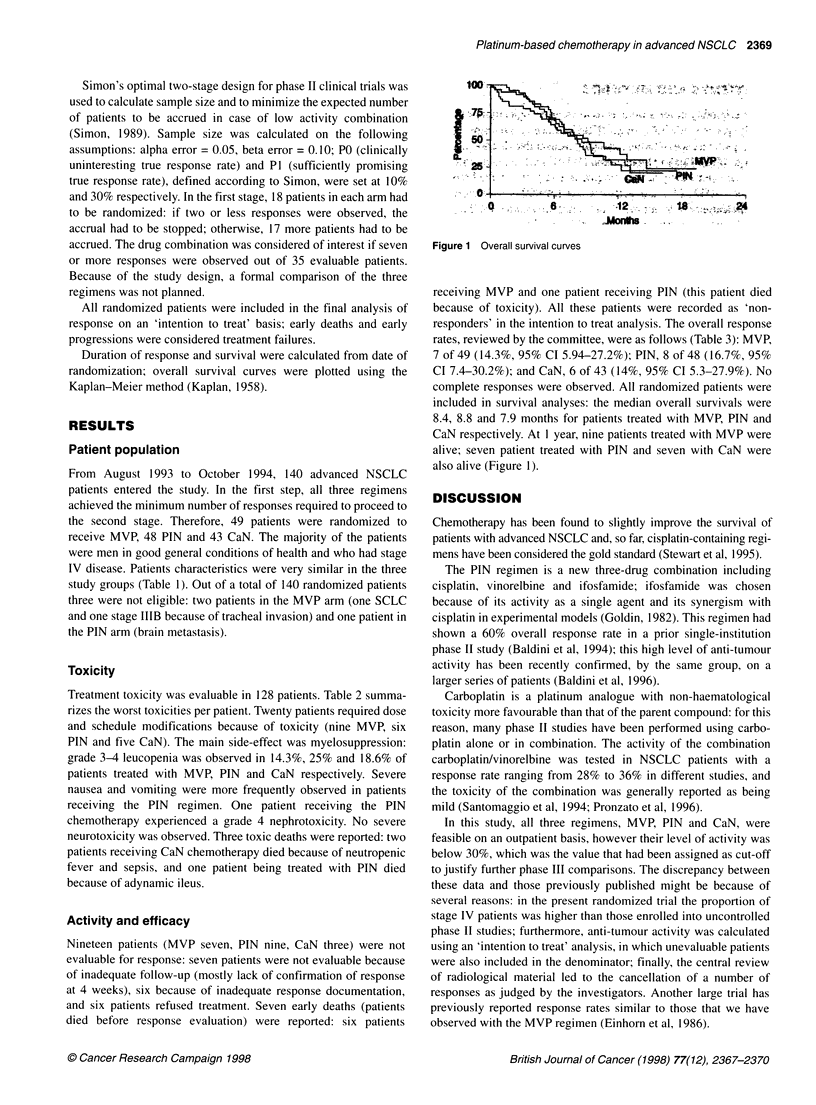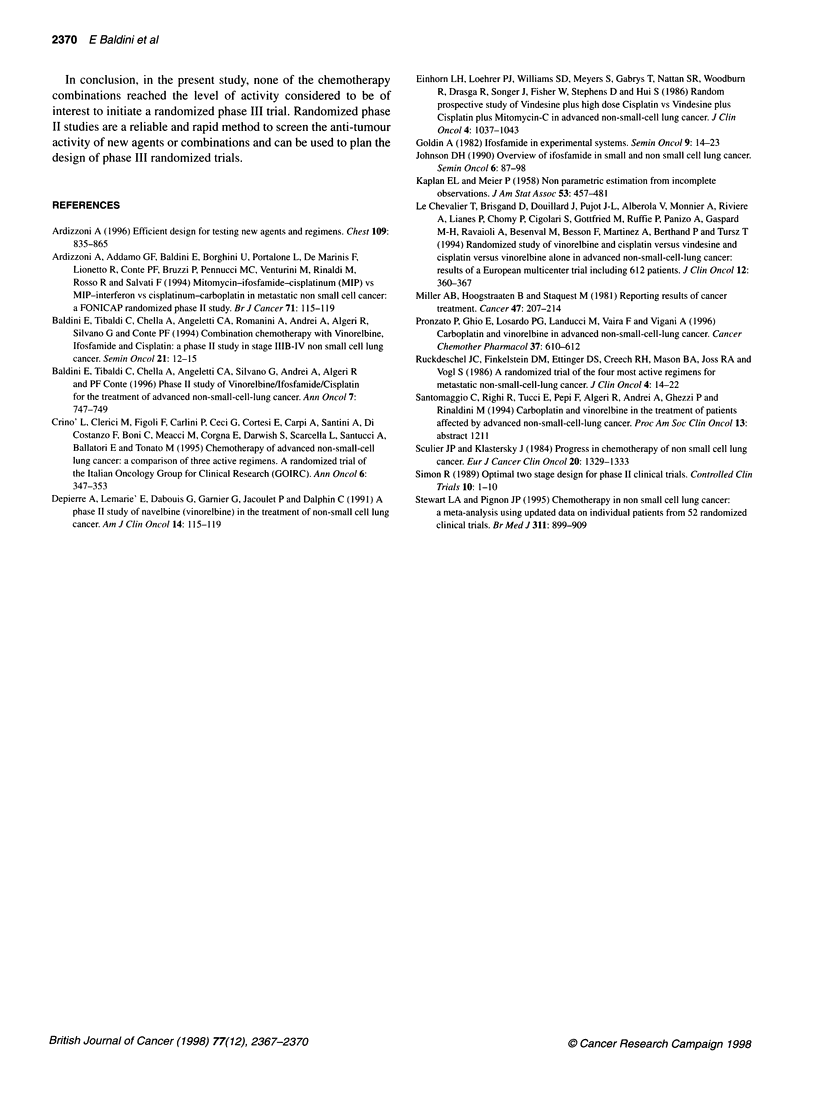# Cisplatin-vindesine-mitomycin (MVP) vs cisplatin-ifosfamide-vinorelbine (PIN) vs carboplatin-vinorelbine (CaN) in patients with advanced non-small-cell lung cancer (NSCLC): a FONICAP randomized phase II study. Italian Lung Cancer Task Force (FONICAP).

**DOI:** 10.1038/bjc.1998.393

**Published:** 1998-06

**Authors:** E. Baldini, C. Tibaldi, A. Ardizzoni, F. Salvati, A. Antilli, L. Portalone, S. Barbera, F. Romano, F. De Marinis, M. R. Migliorino, M. A. Noseda, U. Borghini, M. Crippa, G. Ferrara, M. Raimondi, M. Fioretti, M. Bandera, M. C. Pennucci, G. Galeasso, G. C. Cacciani, G. Lepidini, G. Sunseri, C. Lanfranco, M. Rinaldi, R. Rosso

**Affiliations:** Division of Medical Oncology, Pisa, Italy.

## Abstract

In the present multicentre randomized phase II trial, the activity and toxicity of three platinum-based combination regimens for the treatment of advanced non-small-cell lung cancer (NSCLC) were evaluated. The three regimens were: MVP (mitomycin-C 6 mg m(-2) on day 1, vindesine 3 mg m(-2) on days 1 and 15, and cisplatin 80 mg m(-2) on day 1 every 28 days), PIN (cisplatin 80 mg m(-2) day 1, ifosfamide 3 g m(-2) day 1 and vinorelbine 25 mg m(-2) day 1 and 8 every 21 days) and CaN (carboplatin 350 mg m(-2) day 1 and vinorelbine 25 mg m(-2) days 1 and 8 every 28 days). A total of 140 chemotherapy-naive patients entered the study; 49 patients were treated with MVP, 48 with PIN and 43 with CaN. Sixty-seven per cent of the patients had stage IV disease. Response rates, calculated on an 'intention to treat' basis, were as follows: MVP, 14.3% (95% CI 5.94-27.2%); PIN, 16.7% (95% CI 7.4-30.2%); and CaN, 14% (95% CI 5.3-27.9%). The overall median survivals were 256, 269 and 243 days for patients treated with MVP, PIN and CaN respectively. Myelosuppression was the most frequent toxicity: grade 3-4 leucopenia was observed in 14.3%, 25% and 18.6% of patients treated with MVP, PIN and CaN respectively. This multicentre phase II randomized trial shows that MVP, PIN and CaN can be administered on an outpatient basis with acceptable toxicities. Unfortunately, the three regimens showed an activity significantly lower than that reported in previous single-institution phase II trials.


British Journal of Cancer (1998) 77(12), 2367–2370
© 1998 Cancer Research Campaign

### Cisplatin–vindesine–mitomycin (MVP) vs cisplatin–ifosfamide–vinorelbine (PIN) vs carboplatin–vinorelbine (CaN) in patients with advanced non-small-cell lung cancer (NSCLC): a FONICAP randomized phase II study

**E Baldini[1], C Tibaldi[1], A Ardizzoni[2], F Salvati[3], A Antilli[3], L Portalone[3], S Barbera[4], F Romano[4], F De Marinis[5], MR Migliorino[5], MA Noseda[5], U Borghini[6], M Crippa[6], G Ferrara[7], M Raimondi[7], M Fioretti[8], M Bandera[8], MC Pennucci[2], G Galeasso[9], GC Cacciani[10], G Lepidini[11], G Sunseri[12], C Lanfranco[13], M Rinaldi[14], R Lionetto[2], PF Conte[1] and R Rosso[2] for the Italian Lung Cancer Task Force (FONICAP)**

[1]Division of Medical Oncology, Pisa; [2]National Institute of Cancer Research, Genova; [3]8th Division of Pneumology, Forlanini Hospital, Roma; [4]2nd Division of Pneumology, Cosenza; [5]3rd Division of Pneumology, Forlanini Hospital, Roma; [6]Division of Pneumology, Niguarda Hospital, Milano; [7]1st Division of Pneumology, Palermo; [8]Division of Pneumology, Varese; [9]Division of Medical Oncology, Alba; [10]Division of Pneumology, Parma; [11]Radiotherapy Division, S. Camillo Hospital, Roma; [12]2nd Division of Pneumology, Palermo; [13]Division of Medical Oncology, Asti; [14]Istituto Regina Elena, Roma

**Summary** In the present multicentre randomized phase II trial, the activity and toxicity of three platinum-based combination regimens for the treatment of advanced non-small-cell lung cancer (NSCLC) were evaluated. The three regimens were: MVP (mitomycin-C 6 mg $m^{-2}$ on day 1, vindesine 3 mg $m^{-2}$ on days 1 and 15, and cisplatin 80 mg $m^{-2}$ on day 1 every 28 days), PIN (cisplatin 80 mg $m^{-2}$ day 1, ifosfamide 3 g $m^{-2}$ day 1 and vinorelbine 25 mg $m^{-2}$ day 1 and 8 every 21 days) and CaN (carboplatin 350 mg $m^{-2}$ day 1 and vinorelbine 25 mg $m^{-2}$ days 1 and 8 every 28 days). A total of 140 chemotherapy-naive patients entered the study; 49 patients were treated with MVP, 48 with PIN and 43 with CaN. Sixty-seven per cent of the patients had stage IV disease. Response rates, calculated on an 'intention to treat' basis, were as follows: MVP, 14.3% (95% CI 5.94–27.2%); PIN, 16.7% (95% CI 7.4–30.2%); and CaN, 14% (95% CI 5.3–27.9%). The overall median survivals were 256, 269 and 243 days for patients treated with MVP, PIN and CaN respectively. Myelosuppression was the most frequent toxicity: grade 3–4 leucopenia was observed in 14.3%, 25% and 18.6% of patients treated with MVP, PIN and CaN respectively. This multicentre phase II randomized trial shows that MVP, PIN and CaN can be administered on an outpatient basis with acceptable toxicities. Unfortunately, the three regimens showed an activity significantly lower than that reported in previous single-institution phase II trials.

**Keywords:** platinum-based chemotherapy; advanced non-small-cell lung cancer; randomized phase II study

The treatment of advanced non-small-cell lung cancer (NSCLC) continues to be a challenge to medical oncologists. Several cytotoxic drugs have been extensively investigated, either alone or in combination. As single agents, cisplatin (CDDP), ifosfamide (IFX), mitomycin-C (MMC) and vindesine (VDS) achieve a 5–20% objective response rate in chemotherapy-naive patients (Sculier, 1984; Johnson, 1990). Recently, vinorelbine (VNR) showed interesting activity in a phase II study and in a large randomized trial (Depierre et al, 1991; Le Chevalier et al, 1994). Combination chemotherapy induces a response rate of 20–40%, and some authors have demonstrated that three-drug cisplatin-based regimens are more active and prolong survival compared with two-drug combinations (Crinò et al, 1995). However, it is clear that new active combinations are necessary to improve the prognosis of advanced NSCLC. Unfortunately, in multicentre randomized trials, many promising regimens show an anti-tumour activity lower than that reported in single-institution phase II studies (Ardizzoni et al, 1994; Ardizzoni, 1996). Therefore, the demonstration of a high response rate in a phase II trial is insufficient to justify the implementation of an expensive large randomized trial.

As a result of these findings, the FONICAP group has started a series of consecutive multicentre randomized phase II trials to evaluate the activity of combination regimens previously tested in single-institution phase II studies. The advantage of randomized phase II trials is the minimization of selection bias, which is the main cause of overestimation of response in uncontrolled phase II trials.

In the present study, three regimens were chosen: MVP (mitomycin C, vindesine, cisplatin) was chosen because it was considered to be a reference regimen (Ruckdeschel et al, 1986); PIN (cisplatin, ifosfamide, vinorelbine) and CaN (carboplatin, vinorelbine) had shown interesting activity in phase II trials performed at single institutions participating with the FONICAP Group (Baldini et al, 1996; Pronzato et al, 1996).

*Received 30 May 1997*
*Revised 1 December 1997*
*Accepted 8 December 1997*

*Correspondence to:* A Ardizzoni, Division of Medical Oncology, National Cancer Institute, Largo Rosanna Benzi, 10, 16132 Genova, Italy

### PATIENTS AND METHODS

The eligibility criteria were as follows: histologically or cytologically proven NSCLC, stage IIIB/IV disease; no prior chemotherapy; presence of bidimensionally measurable disease; age ≤ 75 years, World Health Organization (WHO) performance status ≤ 2; normal haematological (haemoglobin ≥ 11 g $dl^{-1}$, white blood cell count ≥ 4000 $\mu l^{-1}$ and platelet count ≥ 100 000 $ul^{-1}$), renal (creatinine clearance > 60 ml $min^{-1}$ and serum creatinine < 1.2 mg $dl^{-1}$) and liver (total bilirubin < 1.2 mg $dl^{-1}$) functions.

The exclusion criteria included the following: active CNS disorder or brain metastasis, cardiovascular disease (cardiac failure, myocardial infarction within the previous 3 months, uncontrolled hypertension or arrhythmias), concomitant neoplasm other than in situ cervical carcinoma or cutaneous basal cell cancer. Patients in relapse after surgery were eligible. Patients previously treated with radiotherapy were eligible if they had other indicator lesions outside the irradiated area.

Pretreatment evaluations included: history and physical examination, white blood cell count and chemistry profile, electrocardiogram (ECG), fibreoptic bronchoscopy, chest radiography, thoracic computerized tomography scan (CT), abdominal CT scan or ultrasound. Bone scan or skeletal radiography and brain CT scan were performed only when clinically indicated. During treatment, white blood cell counts, with differential and platelets, were performed weekly; a physical examination and a chemistry profile were repeated before day 1 of each cycle.

Toxicity was evaluated after each cycle of chemotherapy using standard WHO criteria. A minimum of two cycles of chemotherapy were delivered, unless rapid tumour progression was documented.

Randomization was performed by telephoning the trial office at the National Institute of Cancer Research in Genoa and the patients were assigned to receive one of the following regimens: MVP (MMC 6 mg $m^{-2}$ i.v. on day 1, VDS 3 mg $m^{-2}$ i.v. on days 1 and 15, and CDDP 80 mg $m^{-2}$ i.v. on day 1), PIN (CDDP 80 mg $m^{-2}$ i.v. on day 1, IFX 3 g $m^{-2}$ i.v. on day 1, VNR 25 mg $m^{-2}$ i.v. on days 1 and 8, Mesna 600 mg $m^{-2}$ i.v. before IFX infusion and 1200 mg $m^{-2}$ orally 4 and 8 h after IFX) or CaN (CBDCA 350 mg $m^{-2}$ i.v. on day 1 and VNR 25 mg $m^{-2}$ i.v. on days 1 and 8). MVP and CaN regimens were repeated every 4 weeks; PIN was repeated every 3 weeks. Chemotherapy was administered on day 1 if the white blood cell count was ≥ 4000 μl and platelets ≥ 100 000 ul; in the case of incomplete haematological recovery, chemotherapy was delayed by 1 week; in the case of grade 4 (WHO) haematological toxicity, all the drugs were reduced by 25% in the subsequent courses. In the event of serum creatinine being greater than 2 mg $dl^{-1}$ on day 1, MVP and PIN administration was postponed until normalization, and a 25% dose reduction of CDDP and IFX was applied. The dose of VNR on day 8 was modified according to the absolute neutrophil count (ANC) as follows: grade 2 neutropenia, 25% dose reduction; grade 3 neutropenia, 50% dose reduction; in case of grade 4 neutropenia, VNR was omitted and prophylaxis with oral ciprofloxacin 1 g $d^{-1}$ and Fluconazole 50 mg $d^{-1}$ was started.

Response to treatment was evaluated after two courses of chemotherapy: all target lesions were reassessed with the same technique used at study entry. All responses were checked by a review committee including the study coordinator and an expert radiologist, who were not aware of the type of treatment. Responses were evaluated according to the WHO criteria (Miller et al, 1981).

**Table 1** Patients' characteristics

| | MVP | PIN | CaN |
|---|---|---|---|
| Median age (range) (years) | 62 (37–69) | 64 (45–73) | 61 (47–72) |
| Sex (*n*)[a] | | | |
| Male | 41 (83.6) | 42 (87.5) | 38 (88.3) |
| Female | 8 (16.3) | 6 (12.5) | 5 (11.6) |
| Performance status (*n*)[a] | | | |
| 0 | 17 (34.6) | 22 (45.8) | 20 (46.5) |
| 1 | 22(44.8) | 20 (41.6) | 18 (41.8) |
| 2 | 10 (20.4) | 6 (12.5) | 5 (11.6) |
| Histology (*n*)[a] | | | |
| Squamous cell | 25 (51.0) | 26 (54.1) | 21 (48.8) |
| Adenocarcinoma | 20 (40.8) | 19 (39.5) | 12 (27.9) |
| Large cell | 1 (2.0) | 2 (4.1) | 3 (6.9) |
| Unspecified NSCLC | 3 (6.1) | 1 (2.0) | 7 (16.2) |
| Stage (*n*)[a] | | | |
| IIIb | 15 (30.6) | 18 (37.5) | 14 (32.5) |
| IV | 34 (69.3) | 30 (62.5) | 29 (67.4) |
| Total number | 49 | 48 | 43 |

[a]Numbers in parentheses are percentages.

**Table 2** Toxicities (WHO)

| | MVP (*n* = 45) | | PIN (*n* = 44) | | CaN (*n* = 39) | |
|---|---|---|---|---|---|---|
| | G3 | G4 | G3 | G4 | G3 | G4 |
| Leucopenia (%) | 10.2 | 4.1 | 16.7 | 8.3 | 9.3 | 9.3 |
| Thrombocytopenia (%) | 4.1 | 2.0 | 2.1 | 4.2 | 7.0 | 0 |
| Anaemia (%) | 6.1 | 0 | 10.4 | 0 | 2.3 | 2.3 |
| Nausea and vomiting (%) | 8.2 | 0 | 12.5 | 0 | 2.3 | 0 |
| Nephrotoxicity (%) | 0 | 0 | 0 | 2.1 | 0 | 0 |

**Table 3** Responses to treatment

| Patients | MVP *n* (%) | PIN *n* (%) | CaN *n* (%) |
|---|---|---|---|
| All (intention to treat) | 7/49 (14.3) | 8/48 (16.7) | 6/43 (13.9) |
| Evaluable[a] | 7/40 (17.5) | 8/38 (21.0) | 6/40 (15.0) |
| Adequately treated[b] | 7/34 (20.6) | 8/36 (22.2) | 6/38 (15.8) |

[a]All excluding: unelegible, lost to follow-up, never treated, missing data.
[b]Evaluable excluding: early deaths, toxic deaths.

Patients with complete response (CR), partial response (PR) and stable disease (SD) were treated for a maximum of six courses; patients with progressive disease (PD) were withdrawn from treatment and received supportive care. Informed consent was obtained from all patients according to local institution policies.

### Statistical analysis

A centralized randomization was performed by calling the FONICAP trial office at the National Institute for Cancer Research in Genoa. Allocation to each treatment arm was made using a computer-generated list stratified according to the centre.

© Cancer Research Campaign 1998

Simon's optimal two-stage design for phase II clinical trials was used to calculate sample size and to minimize the expected number of patients to be accrued in case of low activity combination (Simon, 1989). Sample size was calculated on the following assumptions: alpha error = 0.05, beta error = 0.10; P0 (clinically uninteresting true response rate) and P1 (sufficiently promising true response rate), defined according to Simon, were set at 10% and 30% respectively. In the first stage, 18 patients in each arm had to be randomized: if two or less responses were observed, the accrual had to be stopped; otherwise, 17 more patients had to be accrued. The drug combination was considered of interest if seven or more responses were observed out of 35 evaluable patients. Because of the study design, a formal comparison of the three regimens was not planned.

All randomized patients were included in the final analysis of response on an 'intention to treat' basis; early deaths and early progressions were considered treatment failures.

Duration of response and survival were calculated from date of randomization; overall survival curves were plotted using the Kaplan–Meier method (Kaplan, 1958).

### RESULTS

### Patient population

From August 1993 to October 1994, 140 advanced NSCLC patients entered the study. In the first step, all three regimens achieved the minimum number of responses required to proceed to the second stage. Therefore, 49 patients were randomized to receive MVP, 48 PIN and 43 CaN. The majority of the patients were men in good general conditions of health and who had stage IV disease. Patients characteristics were very similar in the three study groups (Table 1). Out of a total of 140 randomized patients three were not eligible: two patients in the MVP arm (one SCLC and one stage IIIB because of tracheal invasion) and one patient in the PIN arm (brain metastasis).

### Toxicity

Treatment toxicity was evaluable in 128 patients. Table 2 summarizes the worst toxicities per patient. Twenty patients required dose and schedule modifications because of toxicity (nine MVP, six PIN and five CaN). The main side-effect was myelosuppression: grade 3–4 leucopenia was observed in 14.3%, 25% and 18.6% of patients treated with MVP, PIN and CaN respectively. Severe nausea and vomiting were more frequently observed in patients receiving the PIN regimen. One patient receiving the PIN chemotherapy experienced a grade 4 nephrotoxicity. No severe neurotoxicity was observed. Three toxic deaths were reported: two patients receiving CaN chemotherapy died because of neutropenic fever and sepsis, and one patient being treated with PIN died because of adynamic ileus.

### Activity and efficacy

Nineteen patients (MVP seven, PIN nine, CaN three) were not evaluable for response: seven patients were not evaluable because of inadequate follow-up (mostly lack of confirmation of response at 4 weeks), six because of inadequate response documentation, and six patients refused treatment. Seven early deaths (patients died before response evaluation) were reported: six patients receiving MVP and one patient receiving PIN (this patient died because of toxicity). All these patients were recorded as 'non-responders' in the intention to treat analysis. The overall response rates, reviewed by the committee, were as follows (Table 3): MVP, 7 of 49 (14.3%, 95% CI 5.94–27.2%); PIN, 8 of 48 (16.7%, 95% CI 7.4–30.2%); and CaN, 6 of 43 (14%, 95% CI 5.3–27.9%). No complete responses were observed. All randomized patients were included in survival analyses: the median overall survivals were 8.4, 8.8 and 7.9 months for patients treated with MVP, PIN and CaN respectively. At 1 year, nine patients treated with MVP were alive; seven patient treated with PIN and seven with CaN were also alive (Figure 1).

**Figure 1** Overall survival curves

### DISCUSSION

Chemotherapy has been found to slightly improve the survival of patients with advanced NSCLC and, so far, cisplatin-containing regimens have been considered the gold standard (Stewart et al, 1995).

The PIN regimen is a new three-drug combination including cisplatin, vinorelbine and ifosfamide; ifosfamide was chosen because of its activity as a single agent and its synergism with cisplatin in experimental models (Goldin, 1982). This regimen had shown a 60% overall response rate in a prior single-institution phase II study (Baldini et al, 1994); this high level of anti-tumour activity has been recently confirmed, by the same group, on a larger series of patients (Baldini et al, 1996).

Carboplatin is a platinum analogue with non-haematological toxicity more favourable than that of the parent compound: for this reason, many phase II studies have been performed using carboplatin alone or in combination. The activity of the combination carboplatin/vinorelbine was tested in NSCLC patients with a response rate ranging from 28% to 36% in different studies, and the toxicity of the combination was generally reported as being mild (Santomaggio et al, 1994; Pronzato et al, 1996).

In this study, all three regimens, MVP, PIN and CaN, were feasible on an outpatient basis, however their level of activity was below 30%, which was the value that had been assigned as cut-off to justify further phase III comparisons. The discrepancy between these data and those previously published might be because of several reasons: in the present randomized trial the proportion of stage IV patients was higher than those enrolled into uncontrolled phase II studies; furthermore, anti-tumour activity was calculated using an 'intention to treat' analysis, in which unevaluable patients were also included in the denominator; finally, the central review of radiological material led to the cancellation of a number of responses as judged by the investigators. Another large trial has previously reported response rates similar to those that we have observed with the MVP regimen (Einhorn et al, 1986).

© Cancer Research Campaign 1998

In conclusion, in the present study, none of the chemotherapy combinations reached the level of activity considered to be of interest to initiate a randomized phase III trial. Randomized phase II studies are a reliable and rapid method to screen the anti-tumour activity of new agents or combinations and can be used to plan the design of phase III randomized trials.

### REFERENCES

Ardizzoni A (1996) Efficient design for testing new agents and regimens. *Chest* **109**: 835–865

Ardizzoni A, Addamo GF, Baldini E, Borghini U, Portalone L, De Marinis F, Lionetto R, Conte PF, Bruzzi P, Pennucci MC, Venturini M, Rinaldi M, Rosso R and Salvati F (1994) Mitomycin–ifosfamide–cisplatinum (MIP) vs MIP–interferon vs cisplatinum–carboplatin in metastatic non small cell cancer: a FONICAP randomized phase II study. *Br J Cancer* **71**: 115–119

Baldini E, Tibaldi C, Chella A, Angeletti CA, Romanini A, Andrei A, Algeri R, Silvano G and Conte PF (1994) Combination chemotherapy with Vinorelbine, Ifosfamide and Cisplatin: a phase II study in stage IIIB-IV non small cell lung cancer. *Semin Oncol* **21**: 12–15

Baldini E, Tibaldi C, Chella A, Angeletti CA, Silvano G, Andrei A, Algeri R and PF Conte (1996) Phase II study of Vinorelbine/Ifosfamide/Cisplatin for the treatment of advanced non-small-cell-lung cancer. *Ann Oncol* **7**: 747–749

Crino' L, Clerici M, Figoli F, Carlini P, Ceci G, Cortesi E, Carpi A, Santini A, Di Costanzo F, Boni C, Meacci M, Corgna E, Darwish S, Scarcella L, Santucci A, Ballatori E and Tonato M (1995) Chemotherapy of advanced non-small-cell lung cancer: a comparison of three active regimens. A randomized trial of the Italian Oncology Group for Clinical Research (GOIRC). *Ann Oncol* **6**: 347–353

Depierre A, Lemarie' E, Dabouis G, Garnier G, Jacoulet P and Dalphin C (1991) A phase II study of navelbine (vinorelbine) in the treatment of non-small cell lung cancer. *Am J Clin Oncol* **14**: 115–119

Einhorn LH, Loehrer PJ, Williams SD, Meyers S, Gabrys T, Nattan SR, Woodburn R, Drasga R, Songer J, Fisher W, Stephens D and Hui S (1986) Random prospective study of Vindesine plus high dose Cisplatin vs Vindesine plus Cisplatin plus Mitomycin-C in advanced non-small-cell lung cancer. *J Clin Oncol* **4**: 1037–1043

Goldin A (1982) Ifosfamide in experimental systems. *Semin Oncol* **9**: 14–23

Johnson DH (1990) Overview of ifosfamide in small and non small cell lung cancer. *Semin Oncol* **6**: 87–98

Kaplan EL and Meier P (1958) Non parametric estimation from incomplete observations. *J Am Stat Assoc* **53**: 457–481

Le Chevalier T, Brisgand D, Douillard J, Pujot J-L, Alberola V, Monnier A, Riviere A, Lianes P, Chomy P, Cigolari S, Gottfried M, Ruffie P, Panizo A, Gaspard M-H, Ravaioli A, Besenval M, Besson F, Martinez A, Berthand P and Tursz T (1994) Randomized study of vinorelbine and cisplatin versus vindesine and cisplatin versus vinorelbine alone in advanced non-small-cell-lung cancer: results of a European multicenter trial including 612 patients. *J Clin Oncol* **12**: 360–367

Miller AB, Hoogstraaten B and Staquest M (1981) Reporting results of cancer treatment. *Cancer* **47**: 207–214

Pronzato P, Ghio E, Losardo PG, Landucci M, Vaira F and Vigani A (1996) Carboplatin and vinorelbine in advanced non-small-cell-lung cancer. *Cancer Chemother Pharmacol* **37**: 610–612

Ruckdeschel JC, Finkelstein DM, Ettinger DS, Creech RH, Mason BA, Joss RA and Vogl S (1986) A randomized trial of the four most active regimens for metastatic non-small-cell-lung cancer. *J Clin Oncol* **4**: 14–22

Santomaggio C, Righi R, Tucci E, Pepi F, Algeri R, Andrei A, Ghezzi P and Rinaldini M (1994) Carboplatin and vinorelbine in the treatment of patients affected by advanced non-small-cell-lung cancer. *Proc Am Soc Clin Oncol* **13**: abstract 1211

Sculier JP and Klastersky J (1984) Progress in chemotherapy of non small cell lung cancer. *Eur J Cancer Clin Oncol* **20**: 1329–1333

Simon R (1989) Optimal two stage design for phase II clinical trials. *Controlled Clin Trials* **10**: 1–10

Stewart LA and Pignon JP (1995) Chemotherapy in non small cell lung cancer: a meta-analysis using updated data on individual patients from 52 randomized clinical trials. *Br Med J* **311**: 899–909

*© Cancer Research Campaign 1998*